# Association of self‐reported religiosity with the development of major depression in multireligious country Japan

**DOI:** 10.1111/pcn.13087

**Published:** 2020-07-05

**Authors:** Daiki Kobayashi, Michael B. First, Takuro Shimbo, Shigenobu Kanba, Yoji Hirano

**Affiliations:** ^1^ Division of General Internal Medicine, Department of Medicine St. Luke's International Hospital Tokyo Japan; ^2^ Department of Epidemiology St. Luke's International University Graduate School of Public Health Tokyo Japan; ^3^ Fujita Heath University Toyoake Japan; ^4^ Department of Psychiatry Columbia University New York USA; ^5^ Ohta Nishinouchi Hospital Koriyama Japan; ^6^ Department of Neuropsychiatry Graduate School of Medical Sciences, Kyushu University Fukuoka Japan; ^7^ Department of Psychiatry Harvard Medical School Boston USA

**Keywords:** Japan, longitudinal study, major depression, multireligious country, religion, spirituality and psychiatry

## Abstract

**Aim:**

In Western Christian countries, religiosity is generally believed to be associated with a lower risk for depression, which is supported by epidemiological evidence. However, the association between religiosity and depression in multireligious countries is unknown. The objective was to evaluate the association between religiosity and subsequent depression in a multireligious population.

**Methods:**

A longitudinal study was conducted in a large hospital in Tokyo, Japan, from 2005 to 2018. All participants who underwent health check‐ups without a prior history of depression or depression at baseline were included. Our outcome was development of major depressive disorder (MDD), which was compared according to the degree of religiosity, adjusting for potential confounders.

**Results:**

Among 67 723 adult participants, those who were more religious tended to be older, female, married, and to have healthier habits but also more medical comorbidities at baseline. During a median follow‐up of 2528 days, 1911 (2.8%) participants developed MDD. Compared to the reference group, religious group participants tended to have higher odds ratios (OR) for developing MDD in a dose‐dependent manner. Among them, the extremely religious group (OR, 1.51; 95% confidence interval [CI], 1.28–1.78) and the moderately religious group (OR, 1.30; 95% CI, 1.14–1.49) were statistically associated with increased development of MDD compared to the not‐religious‐at‐all group. Those who had increased their religiosity from baseline had statistically lower development of MDD (OR, 0.85; 95% CI, 0.75–0.97) compared to those who remained in the same degree of religiosity from baseline.

**Conclusion:**

Religiosity was associated with future MDD in a dose‐dependent manner in a multireligious population, which was in the opposite direction from that seen in previous Western longitudinal studies.

Religiosity is known to be associated with health outcomes, including both physical and mental health, and longevity in a healthy population.^1^ A previous meta‐analytic review reported that religious involvement was significantly associated with longevity.^2^ Another meta‐analysis also showed lower pooled all‐cause mortality and cardiovascular mortality risk among those with higher religiosity/spirituality in a healthy population.^3^ Religiosity was also inversely associated with the future development of diabetes based on our previous study among people without cardiovascular diseases.^4^

Evidence is inconsistent in terms of the association between religiosity and depression. A previous meta‐analysis reported that greater religiosity was associated with lower incidence of mental health.^5^ Another meta‐analysis, mainly composed of cross‐sectional studies, also found a similar relationship between religiosity and depression, a relationship that was enhanced in a highly stressed subgroup, suggesting buffering effects of religiosity on stress.^6^ In contrast, one international longitudinal study reported that religious people had higher risk of major depression.^7^ Given that both positive and negative association of religiosity to subsequent depression have been reported, additional studies examining the relationship of religiosity to depression are needed.

Notably, most of these studies were conducted cross‐sectionally and also in Christianity‐dominant countries, such as the USA and Europe.^8^ The perspective that religiosity is associated with health in a healthy population is consistent with the hypotheses that religiosity reduces stress^9^ and promotes personal independence^10^ and cultural norms that emphasize individual rights and welfare. However, these perspectives stem from Western cultures, and it is unclear whether they are applicable to non‐Western cultures, especially Oriental cultures where cultural norms focus on social obligations.

The Japanese have unique situations for both religiosity and depression. Approximately 59.7% of the Japanese population belonged to the Shinto religion, 36.1% to Buddhism, 0.7% to Christianity, and 3.6% to other religions based on a national survey by the Japanese Ministry of Health, Labour and Welfare in 2018. (Note that because this survey was collected based on religious organizations' reports, some people may have been overcounted and the number of believers may have been overestimated. In fact, the total number of believers was more than 180 million, although the current number of the Japanese population is approximately 120 million.)^11^ However, most Japanese enjoy and practice religious customs and rituals from a variety of religious cultures,^12^ cerebrating Christmas, visiting Shinto shrines on New Year's Day, and commemorating their dead in Buddhist temples. Another study reported that only 10% of the Japanese population considered religion to be very important to them.^13^ Because of the absence of strict scriptures and regular religious gatherings in Shinto and Buddhism, social cohesion may be weak compared to other religions whose practices promote social cohesion. In addition, compared to many Christian groups that consider smoking cigarettes and drinking alcohol to be sinful and thus discourage unhealthier lifestyles, the lack of such prohibitions under Shinto and Buddhism^4^ might lead to comparatively unhealthier lifestyles. Considering these situations, the Japanese population may be less religious, and religiosity may have less impact on Japanese health levels.

Major depression in Japan also has unique characteristics. Japan ranked 58th on the 2019 World Happiness Report, which was the lowest rank among G7 countries and lower than Organisation for Economic Co‐operation and Development countries.^14^ Furthermore, Japan ranked 13th in a 2016 World Health Organization report on suicide rates, which might reflect lower happiness levels.^15^ Importantly, however, the prevalence of mental disorders, including depression, was said to be lower in Japan than in Western countries.^16^, ^17^ The discrepancy between happiness and suicide rate, and prevalence of depression may be due to Japanese citizens hesitating to consult with psychologists or psychiatrists about their mental disorders owing to their stigma against mental problems. In fact, a relatively large number of people in Japan (a lower happiness country) seem to have severe psychological distress, which may lead to suicidal ideation, but the rate of mental health service use among those who have psychological distress without a history of depression is much lower than that of people with a history of depression.^18^ In other words, despite severe psychological dysfunctions, many people who have psychological distress are quite unlikely to see psychiatrists.

Given that Japan has a unique situation of being less religious overall, having mixed religious cultures, and having a lower prevalence of depression compared to Western countries, we sought more evidence regarding the association between religiosity and subsequent depression. The aim of this study was to evaluate the association between religiosity and subsequent depression in a mixed religious population.

## Methods

A retrospective longitudinal study was conducted at St. Luke's International Hospital, a large teaching hospital in Tokyo, Japan, from 2005 to 2018, at which, despite its name, only 5% of outpatients are foreigners. We included all participants who underwent voluntary health check‐ups at the Center for Preventive Medicine in the hospital from 2005 to 2010. Those who had prior history of major depression based on participants' self‐report or information from the medical records at the hospital were excluded. Our primary outcome was incidence of major depression during follow‐up periods up to 31 December 2018. We compared the incidence of major depression by self‐reported degree of religiosity, after adjusting for potential confounders.

The Ethics Committee Institutional Review Board at the hospital approved this study (approved number:18‐R203: Comprehensive approvals for studies about social habits). Because this was a retrospective study, written consent was waived by the ethical committee. However, those who declared not to participate in this study by noticing the research information on the hospital website were excluded (opt‐out).

### Outcomes

Our primary outcome was the incidence of major depression based on the information from medical records at the hospital and participants' self‐reports. The diagnosis of major depression at the hospital was made by an experienced physician based on the DSM‐IV.^19^, ^20^ The information of the diagnosis was extracted based on ICD‐10 codes from electronic medical records. In addition, participants reported their current and past medical histories by responding to questionnaires at each visit. Participants were followed for the development of major depression up to 31 December 2018.

### Degree of religiosity

All participants who underwent the health check‐ups were asked about their religiosity by responding to the following self‐report question: “Are you religious?” Participants were asked to rate their religiosity on an ascending 4‐point Likert scale with rating options: *not at all religious*, *slightly religious*, *moderately religious*, or *extremely religious*. We divided participants into four groups accordingly. The questionnaire was administered at each visit and the degree of religiosity was represented as a time‐dependent variable because some may have changed their degree of religiosity over time. We defined the “not‐at‐all‐religious” group as the reference group. In addition, we categorized participants into three groups based on how their degree of religiosity had changed from their first visit to their last follow‐up: *decreased*, *no change*, and *increased*.

### Covariates

As potential covariates, we obtained information about participants' demographic characteristics, health habits, and medical histories. Information about participants' age, sex, body mass index (BMI), and marital status was obtained at each visit as part of questionnaires in the health check‐ups. BMI was calculated with measured height and weight at health check‐ups and categorized into *underweight* (BMI < 18.5 kg/m^2^), *normal* (18.5–24.9 kg/m^2^), or *overweight/obese* (≥25.0 kg/m^2^) based on Asian criteria.^21^ Health habits involved alcohol consumption (*abstainer*, *occasional drinker*, or *regular drinker*), smoking status (*never*, *former*, or *current*) and exercise habit (*almost none*, *1–2 times a week*, *3–5 times a week*, or *almost every day*). Medical histories included current history of diabetes, hypertension, dyslipidemia, and any types of cancer, and past history of any types of cancer based on participants' self‐reports. All these data were also obtained at each visit and considered as time‐dependent covariates.

### Statistical methods

Participants' baseline characteristics and future outcomes were compared according to their baseline degree of religiosity. Then, multivariable logistic analyses with generalized estimating equation adjusting for potential covariates were used for the development of major depression. When we applied generalized estimating equation, binomial distribution and the logit link function were used. To confirm the results, we adjusted different covariates in the different models: Model 1 was adjusted for time variable, and participant's age and sex; Model 2 was adjusted for health habits (smoking, alcohol consumption, and exercise) and BMI in addition to covariates in Model 1; Model 3 was adjusted for marital status in addition to covariates in Model 2; and Model 4 was adjusted for medical histories (current hypertension, diabetes, dyslipidemia and any cancer; and any past cancer) in addition to covariates in Model 3. As a subanalysis, we performed similar analyses stratifying by sex. In addition, we conducted three sensitivity analyses: (i) with the data focusing on major depression that required antidepressant treatment in order to evaluate the association with severe depression and focusing on a more accurate diagnosis of major depression; (ii) with the data excluding major depression developed within 2 years from baseline to exclude the effects of potential major depression at baseline; and (iii) with the analyses by time‐independent Cox proportional hazard model to confirm robustness. Moreover, change of religiosity during the study period (became less religious from baseline religiosity to last observed religiosity; no change; became more religious from baseline religiosity to last observed religiosity) was included in the analyses to investigate how the change of religiosity was associated with depression.

All analyses were performed using stata 14 in 2019 (STATA Corp., College Station, TX, USA).

## Results

We included a total of 67 723 participants during the study period. The mean age of our participants was 46.3 years (SD: 12.3 years), 33 893 (50.1%) were male, and 48 051 (71.0%) were married. Participants who were more religious tended to be older, female, and married (Table 1). In terms of health habits, participants tended to be healthier, smoked less, drank less alcohol, and engaged in more regular exercise as they were more religious. However, more religious people also tended to be more overweight/obese and tended to have more medical comorbidities than those who were less religious.

**Table 1:** Baseline characteristics and outcome according to baseline religiosity

	Religiosity				Total	
	Not religious at all	Slightly religious	Moderately religious	Extremely religious		
	(*n* = 16,473)	(*n* = 26,057)	(*n* = 18,522)	(*n* = 6671)	(*n* = 67,723)	*P* value
Outcome, *n* (%)						
Depression	398 (2.4)	670 (2.6)	600 (3.2)	243 (3.6)	1911 (2.8)	<0.01
Demographics						
Age, years (SD)	41.8 (11.2)	45.4 (11.5)	49.4 (12.2)	52.4 (13.1)	46.3 (12.3)	<0.01
Male, *n* (%)	9020 (54.8)	13,664 (52.4)	8331 (45.0)	2878 (43.1)	33,893 (50.1)	<0.01
Married, *n* (%)	10,636 (64.6)	18,603 (71.4)	13,777 (74.4)	5035 (75.5)	48,051 (71.0)	<0.01
Change of religiosity, *n* (%)						<0.01
Decreased overtime	—	3366 (12.9)	3157 (17.0)	1307 (19.6)	7830 (11.6)	
No change	12,021 (73.0)	18,410 (70.7)	13,722 (74.1)	5364 (80.4)	49,517 (73.1)	
Increased overtime	4452 (27.0)	4281 (16.4)	1643 (8.9)	—	10,376 (15.3)	
Body mass index, *n* (%)						<0.01
Underweight (<18.5 kg/m^2^)	1694 (10.3)	2453 (9.4)	1723 (9.3)	594 (8.9)	6463 (9.5)	
Normal (18.5–24.9 kg/m^2^)	11,753 (71.4)	18,646 (71.6)	13,057 (70.5)	4664 (69.9)	48,120 (71.1)	
Overweight/obese (≥25.0 kg/m^2^)	3024 (18.4)	4959 (19.0)	3742 (20.2)	1413 (21.2)	13,138 (19.4)	
Follow‐up, days (interquartile range)	2381 (837–3858)	2498 (927–4001)	2615 (1071–4082)	2569 (1069–4078)	2528 (974–4007)	<0.01
Health habits, *n* (%)						
Alcohol						<0.01
Abstainer	6195 (37.6)	9488 (36.4)	7505 (40.5)	3183 (47.7)	26,371 (38.9)	
Occasional	2781 (16.9)	4777 (18.3)	3345 (18.1)	1131 (17.0)	12,034 (17.8)	
Regular	7497 (45.5)	11,792 (45.3)	7672 (41.4)	2357 (35.3)	29,318 (43.3)	
Smoking						<0.01
Never	9389 (57.0)	15,496 (59.5)	11,645 (62.9)	4385 (65.7)	40,915 (60.4)	
Former	3265 (19.8)	5924 (22.7)	4344 (23.5)	1482 (22.2)	15,015 (22.2)	
Current	3819 (23.2)	4637 (17.8)	2533 (13.7)	804 (12.1)	11,793 (17.4)	
Exercise						<0.01
Almost none	7333 (44.5)	9861 (37.8)	6094 (32.9)	2111 (31.6)	25,399 (37.5)	
1–2 times per week	5775 (35.1)	10,150 (39.0)	7021 (37.9)	2295 (34.4)	25,241 (37.3)	
3–5 times per week	2043 (12.4)	3793 (14.6)	3350 (18.1)	1248 (18.7)	10,434 (15.4)	
Almost all days	1322 (8.0)	2253 (8.7)	2057 (11.1)	1017 (15.3)	6649 (9.8)	
Comorbidities, *n* (%)						
Hypertension	864 (5.2)	1839 (7.1)	1690 (9.1)	785 (11.8)	5178 (7.7)	<0.01
Diabetes	287 (1.7)	584 (2.2)	523 (2.8)	281 (4.2)	1675 (2.5)	<0.01
Dyslipidemia	446 (2.7)	1028 (4.0)	1053 (5.7)	457 (6.9)	2984 (4.4)	<0.01
Current history of any cancer	283 (1.7)	648 (2.5)	635 (3.4)	272 (4.1)	1838 (2.7)	<0.01
Past history of any cancer	135 (0.8)	275 (1.1)	268 (1.5)	135 (2.0)	813 (1.2)	<0.01

**Figure 1:**
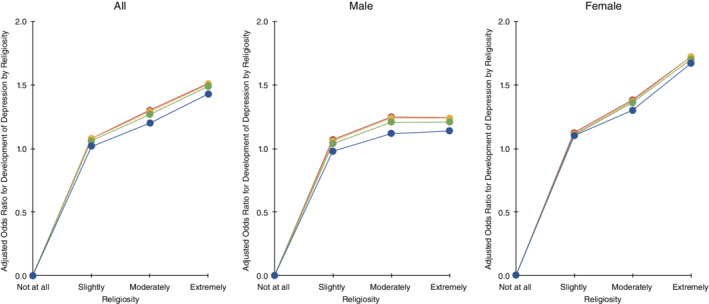
Adjusted odds ratio for development of depression by religiosity at baseline: Longitudinal analyses. Model 1 (blue) was adjusted for time variable, and participant's age and sex. Model 2 (green) was adjusted for health habits (smoking, alcohol consumption, and exercise) and body mass index in addition to covariates in Model 1. Model 3 (yellow) was adjusted for marital status in addition to covariates in Model 2. Model 4 (red) was adjusted for medical histories (current hypertension, diabetes, dyslipidemia, and any cancer; and any past cancer) in addition to covariates in Model 3.

During the median follow‐up of 2528 days (interquartile range: 974–4007 days), 1911 (2.8%) participants developed major depression. The median number of follow‐up visits was six (interquartile range: 3–10). Table 2 shows the odds ratios (OR) for development of major depression by degree of religiosity from multivariate longitudinal analyses. Compared to the reference group (not religious at all), the more religious groups tended to have higher OR for development of major depression in a dose‐dependent manner (Fig. 1). Among them, the extremely religious group (OR, 1.51; 95% confidence interval [CI], 1.28–1.78 in Model 4) and moderately religious group (OR, 1.30; 95%CI, 1.14–1.49 in Model 4) were statistically associated with increased development of major depression compared to the not‐religious‐at‐all group. In terms of the association between history of cancer and the development of major depression from multivariate analyses, cancer patients tended to have a higher incidence of major depression, but this was not statistically significant (current history of any cancer: OR, 1.19; 95%CI, 0.99–1.43; past history of any cancer: OR, 1.21; 95%CI, 0.94–1.55 in Model 4).

**Table 2:** Adjusted odds ratios for development of depression according to religiosity from longitudinal analyses

	Adjusted odds ratio (95% confidence interval)			
	Religiosity			
	Model 1	Model 2	Model 3	Model 4
Not religious at all	Reference	Reference	Reference	Reference
Slightly religious	1.02 (0.89–1.15)	1.06 (0.93–1.21)	1.08 (0.95–1.22)	1.08 (0.95–1.23)
Moderately religious	**1.20 (1.05–1.37)**	**1.27 (1.12–1.46)**	**1.29 (1.13–1.48)**	**1.30 (1.14–1.49)**
Extremely religious	**1.43 (1.22–1.68)**	**1.49 (1.26–1.75)**	**1.51 (1.28–1.78)**	**1.51 (1.28–1.78)**

Model 1 was adjusted for time variable, age, and sex. Model 2 was adjusted for health habits (smoking, alcohol consumption, and exercise) and body mass index in addition to the covariates in Model 1. Model 3 was adjusted for marital status in addition to the covariates in Model 2. Model 4 was adjusted for medical history (current hypertension, diabetes, dyslipidemia, and any cancer, and any past cancer) in addition to the covariates in Model 3.

Numbers in bold indicate *P* < 0.05.

In our subanalysis stratified by sex, the findings that the more religious groups had higher OR of major depression were still observed. However, the dose‐dependent association was more obvious and stronger in female patients compared to male (Table 3; Fig. 1).

**Table 3:** Adjusted odds ratios for development of depression according to religiosity from longitudinal analyses

	Adjusted odds ratio (95% confidence interval)			
	Religiosity			
	Model 1	Model 2	Model 3	Model 4
Female				
Not religious at all	Reference	Reference	Reference	Reference
Slightly religious	1.10 (0.89–1.29)	1.10 (0.91–1.33)	1.11 (0.92–1.34)	1.12 (0.93–1.35)
Moderately religious	**1.30 (1.08–1.56)**	**1.36 (1.13–1.63)**	**1.37 (1.14–1.65)**	**1.38 (1.15–1.66)**
Extremely religious	**1.67 (1.35–2.07)**	**1.70 (1.37–2.11)**	**1.72 (1.39–2.13)**	**1.72 (1.39–2.13)**
Male				
Not religious at all	Reference	Reference	Reference	Reference
Slightly religious	0.98 (0.82–1.17)	1.04 (0.87–1.25)	1.06 (0.89–1.27)	1.07 (0.96–1.28)
Moderately religious	1.12 (0.92–1.36)	1.21 (0.99–1.47)	**1.24 (1.02–1.51)**	**1.25 (1.03–1.52)**
Extremely religious	1.14 (0.88–1.48)	1.21 (0.94–1.58)	1.24 (0.95–1.61)	1.24 (0.96–1.62)

Model 1 was adjusted for time variable, age, and sex. Model 2 was adjusted for health habits (smoking, alcohol consumption, and exercise) and body mass index in addition to the covariates in Model 1. Model 3 was adjusted for marital status in addition to the covariates in Model 2. Model 4 was adjusted for medical history (current hypertension, diabetes, dyslipidemia, and any cancer, and any past cancer) in addition to the covariates in Model 3.

Numbers in bold indicate *P* < 0.05.

Our sensitivity analysis with the major depression that required antidepressant treatment showed a similar association that incidence of major depression increased with increased religiosity. However, the extremely religious group had a lower OR of major depression compared to the moderately religious group, and did not have statistically higher OR compared to the not‐religious‐at‐all group (Supplement S1). When we excluded the development of major depression within 2 years from baseline, the results were still very similar to the results in the main analyses (Supplement S2). The results of another sensitivity analysis with the analyses by time‐independent Cox proportional hazard model are shown in Supplement S3. Dose–response associations between degree of religiosity and development of major depression were still observed among all participants and female participants, but not among male participants.

When focusing on the change of religiosity, those who had increased their religiosity from baseline had statistically lower development of major depression (OR, 0.85; 95%CI, 0.75–0.97) compared to those who remained in the same degree of religiosity from baseline. However, those who had decreased their religiosity from baseline had similar risk of major depression compared to those who remained in the same degree of religiosity from baseline (OR, 0.91; 95%CI, 0.78–1.06).

## Discussion

Our longitudinal study demonstrated that religiosity at baseline was associated with future major depression in a dose–response manner in a multireligious population. In addition, this dose‐dependent association was stronger in female patients compared to male. Interestingly, however, those who had increased their religiosity after the initial evaluation had lower risk of major depression.

As opposed to many European studies that found an inverse association between degree of religiosity and subsequent major depression after adjusting for potential covariates,^6^, ^22^, ^23^ our study showed a positive association between degree of religiosity and subsequent major depression, even adjusting for similar covariates. We could interpret this result as consistent with the hypothesis that religiosity might attract that portion of the Japanese population who had serious burden or stress on their own lives. The Japanese population, even those who belong to Buddhism, Shinto, Christianity, or other religions, usually pray or rely on their gods when they are in trouble, want to improve their luck, or get their wishes fulfilled. In other words, they usually attend or involve religious things when they are in need of doing so, rather than having a regular involvement in them. Indeed, more than 25% of our study population reported that they changed their degree of religiosity during the study period. Importantly, our hypothesis was supported by a previous study in the Japanese population, reporting that survivors after traumatic events tended to have spiritual change.^24^

Although many studies from Western countries were supportive of the negative association between degree of religiosity and mental problems, a few studies showed unique associations. For instance, a previous meta‐analysis also found positive correlation between religiosity and mental health, including major depression.^5^ A study from France showed a negative association between degree of religiosity and suicide rate,^25^ whereas one from the Netherlands showed a U‐shape association between degree of religiosity and depressive symptoms.^26^ Although the reasons for such discrepancies are unknown, the potential mechanisms/reasons for the association in Japan may be shared with those in the Netherlands.

We also considered intrinsic and extrinsic religiosity as a potential explanation for our finding.^27^ Batson and Raynor‐Prince have advocated that people with extrinsic religiousness consider religion as a method to achieve non‐religious goals and view it as a means to an end, whereas people with intrinsic religiousness consider religion as that end.^28^ In other words, extrinsic religiosity is a source of social connection to others and personal benefits, whereas intrinsic religiosity is a core value of the person.^29^ A previous study reported that Japanese religions appeared to be rooted in extrinsic motivation from a Judeo‐Christian point of view because participants tried to obtain benefits for their own lives from the religions.^30^ Japanese people who had strong extrinsic religiosity may label themselves as very religious, resulting in a positive association with major depression. People with extrinsic orientation reportedly may have more stress due to seeking extrinsic goals, such as fame, attractiveness, and wealth.^31^ As a result, extrinsic religiosity was said to be associated with anxiety based on a previous study.^32^ In fact, a previous study that compared the association between religiosity and psychological well‐being across countries showed a negative correlation in the Japanese sample, although it was not statistically significant. Therefore, one of the potential explanations for the association between degree of religiosity and development of major depression in the Japanese population may be the existence of extrinsic religiosity.

As regards subanalyses by sex, positive associations between degree of religiosity and subsequent major depression were more obvious among females. This phenomenon may be related to sex‐role socialization in Japan, where females are more likely to help others.^33^ Under these sex roles, Japanese females usually have more burden from housework and less benefit from working outside.^34^ This sex‐role socialization may cause psychological distress and strong dependence on religions in Japanese females.

In the subanalysis considering changes of degree of religiosity, those who became more religious during the study period had significantly lower risk of future depression compared to those who were still in the same degree of religiosity. This result may be interpreted as increased religiosity having protective effects against major depression to an extent even in the Japanese population. However, because more religious people had higher OR of subsequent major depression even after including the change of degree of religiosity, the magnitude of the preventive effects against major depression could be smaller than that of the association between degree of religiosity and depression.

Our study has some limitations. First, we did not have detailed information about degree of religiosity other than self‐report. In addition, a simple question (“Are you religious?”) may not sufficiently evaluate one's degree of religiosity. However, it would be difficult to obtain objective degrees of religiosity across religions in Japan due to differences in religious cultures. For instance, Shinto and Buddhism do not have regular religious gatherings like the Christian Mass. Similarly, our dataset did not specify the type of religion, as this dataset stemmed from participants during voluntary health check‐ups. One may argue that religion type may have some effects on the magnitude of the association, which is desirable for future work. Another limitation was that major depression may not have been consistently or validly diagnosed by all of the physicians.^35^ The diagnosis of major depression is sometimes complicated, even for very experienced physicians, and some were patient‐reported diagnoses, so this diagnosis of major depression may not have been accurate. Although our sensitivity analysis focusing on patients with major depression who required medication may mitigate potential inaccuracy, it does not eliminate it so that diagnostic accuracy may still have had a slight effect on the results. In addition, we did not obtain data about the severity of major depression. Therefore, we conducted a sensitivity analysis with the data focusing on major depression that required antidepressant treatment. Moreover, although we excluded patients with major depression at baseline, our data include neither the information about participants’ personality traits nor psychological conditions at baseline. For example, because people with higher neuroticism or harm avoidance are known to have a higher risk of major depression,^36^ this information may confound the association between religiosity and development of major depression, which would be an interesting topic for future work. In addition, we did not obtain information about participants' stressful life experiences, such as traumatic events, before and during the study. This information could be another potential confounder to the association. Given that St. Luke's International Hospital was founded by a missionary in accordance with his Christian love, there is a possibility that our patients' religion profiles may be slightly different from those of other hospitals. Nevertheless, because Christian participants would have a negative association between religiosity and the development of major depression based on previous European studies, an increased percentage of the Christian participants in this study should have had the opposite effect on our findings. However, this is not the case with our study, where we found a significant positive association between religiosity at baseline and the development of major depression. Finally, as the hospital is located in central Tokyo, our study participants may not be generalized to the entire Japanese population.

### Conclusion

Our large‐cohort longitudinal study demonstrated that religiosity at the baseline was associated with future major depression in a dose–response manner in a multireligious population, which was in the opposite direction from that seen in previous Western longitudinal studies. However, those who had increased their religiosity afterwards had lower risk of major depression. Thus, in contrast with Western Christian culture, the Japanese multireligious culture may have a different impact on the religiosity–depression association. Our results suggest a new perspective for the religiosity–depression association with the notion that cultural and religious diversity must be taken into consideration.

## Disclosure statement

M.B.F. is on the faculty of the Lundbeck International Neuroscience Foundation. All other authors declare they have no conflicts of interest.

## Author contributions

D.K. and Y.H. contributed to conception and design. D.K. and T.S. processed the original dataset. D.K. performed the analyses and prepared the first draft of the manuscript. Y.H. and M.B.F. supervised the analyses. M.B.F. and S.K. revised the manuscript for intellectual content. D.K. and Y.H. critically revised the manuscript for important intellectual content. All authors gave final approval and agree to be accountable for all aspects of the work in ensuring that questions relating to the accuracy or integrity of any part of the work are appropriately investigated and resolved.

## Supporting information


**Supplement S1**. Adjusted odds ratios for development of depression that required antidepressant treatment according to religiosity from longitudinal analyses.

**Supplement S2**. Adjusted odds ratios for development of depression diagnosed after 2 years from baseline according to religiosity from longitudinal analyses.

**Supplement S3**. Adjusted hazard ratios for development of depression according to religiosity from time‐independent Cox proportional‐hazards model.
