# Supplementary material for: Association of self‐reported religiosity with the development of major depression in multireligious country Japan
Source: Psychiatry Clin Neurosci. 2020 Jul 5;74(10):535–41. doi: 10.1111/pcn.13087 (PMC7586836; doi:10.1111/pcn.13087)
Supplement: Supplementary file 1 — Supplement S1. Adjusted odds ratios for development of depression that required antidepressant treatment according to religiosity from longitudinal analyses. [file PCN-74-535-s001.docx]

Supplement 1. Adjusted odds ratio for development of depression that required anti-depressant treatment, by religiosity from longitudinal analyses

|  | Adjusted odds ratio  (95% confidence interval) | | | |
| --- | --- | --- | --- | --- |
|  | Religiosity | | | |
|  | Model 1 | Model 2 | Model 3 | Model 4 |
| Not religious at all | reference | reference | reference | reference |
| Slightly religious | 0.98 (0.81 - 1.17) | 1.02 (0.85 - 1.23) | 1.03 (0.86 - 1.24) | 1.03 (0.86 - 1.24) |
| Moderately religious | **1.30 (1.07 – 1.57)** | **1.38 (1.14 – 1.67)** | **1.39 (1.15 – 1.68)** | **1.39 (1.14 – 1.68)** |
| Extremely religious | 1.24 (0.95 – 1.60) | 1.28 (0.98 – 1.66) | 1.29 (0.99 – 1.67) | 1.28 (0.99 – 1.66) |

Model 1 was adjusted for time variable, age, and sex. Model 2 wad adjusted for health habits (smoking, alcohol consumption and exercise) and body mass index in addition to the covariates in Model 1. Model 3 was adjusted for marital status in addition to the covariates in Model 2. Model 4 was adjusted for medical history (current hypertension, diabetes, dyslipidemia, and any cancer, and any past cancer) in addition to the covariates in Model 3.

Numbers in bold indicate p <0.05.
