# Supplementary material for: Association of self‐reported religiosity with the development of major depression in multireligious country Japan
Source: Psychiatry Clin Neurosci. 2020 Jul 5;74(10):535–41. doi: 10.1111/pcn.13087 (PMC7586836; doi:10.1111/pcn.13087)
Supplement: Supplementary file 2 — Supplement S2. Adjusted odds ratios for development of depression diagnosed after 2 years from baseline according to religiosity from longitudinal analyses. [file PCN-74-535-s002.docx]

Supplement 2. Adjusted odds ratio for development of depression diagnosed after 2 years from baseline, by religiosity from longitudinal analyses

|  | Adjusted odds ratio  (95% confidence interval) | | | |
| --- | --- | --- | --- | --- |
|  | Religiosity | | | |
|  | Model 1 | Model 2 | Model 3 | Model 4 |
| Not religious at all | reference | reference | reference | reference |
| Slightly religious | 1.03 (0.89 - 1.20) | 1.08 (0.93 - 1.26) | 1.09 (0.94 - 1.28) | 1.10 (0.94 - 1.28) |
| Moderately religious | **1.22 (1.05 – 1.43)** | **1.30 (1.11 – 1.52)** | **1.32 (1.13 – 1.55)** | **1.33 (1.14 – 1.56)** |
| Extremely religious | **1.38 (1.14 – 1.67)** | **1.43 (1.18 – 1.74)** | **1.45 (1.20 – 1.76)** | **1.45 (1.20 – 1.76)** |

Model 1 was adjusted for time variable, age, and sex. Model 2 wad adjusted for health habits (smoking, alcohol consumption, and exercise) and body mass index in addition to the covariates in Model 1. Model 3 was adjusted for marital status in addition to the covariates in Model 2. Model 4 was adjusted for medical history (current hypertension, diabetes, dyslipidemia, and any cancer, and any past cancer) in addition to the covariates in Model 3.

Numbers in bold indicate p <0.05.
