# Supplementary material for: Association of self‐reported religiosity with the development of major depression in multireligious country Japan
Source: Psychiatry Clin Neurosci. 2020 Jul 5;74(10):535–41. doi: 10.1111/pcn.13087 (PMC7586836; doi:10.1111/pcn.13087)
Supplement: Supplementary file 3 — Supplement S3. Adjusted hazard ratios for development of depression according to religiosity from time‐independent Cox proportional‐hazards model. [file PCN-74-535-s003.docx]

Supplement 3. Adjusted hazard ratios for development of depression, by religiosity from time-independent Cox proportional-hazards model

|  | Adjusted hazard ratio  (95% confidence interval) | | | |
| --- | --- | --- | --- | --- |
|  | Religiosity | | | |
| Total participants | Model 1 | Model 2 | Model 3 | Model 4 |
| Not religious at all | reference | reference | reference | reference |
| Slightly religious | 0.99 (0.88 - 1.12) | 1.02 (0.90 - 1.15) | 1.04 (0.91 - 1.17) | 1.04 (0.91 - 1.17) |
| Moderately religious | 1.14 (0.99 – 1.30) | **1.17 (1.03 – 1.34)** | **1.20 (1.05 – 1.36)** | **1.20 (1.05 – 1.37)** |
| Extremely religious | **1.24 (1.06 – 1.47)** | **1.27 (1.08 – 1.50)** | **1.29 (1.09 – 1.52)** | **1.29 (1.09 – 1.52)** |
| Female |  |  |  |  |
| Not religious at all | reference | reference | reference | reference |
| Slightly religious | 1.06 (0.89 - 1.27) | 1.09 (0.91 - 1.30) | 1.10 (0.92 - 1.32) | 1.10 (0.92 - 1.32) |
| Moderately religious | **1.25 (1.05 – 1.50)** | **1.29 (1.08 – 1.55)** | **1.31 (1.09 – 1.57)** | **1.32 (1.10 – 1.58)** |
| Extremely religious | **1.52 (1.23 – 1.89** | **1.56 (1.26 – 1.94)** | **1.57 (1.27 – 1.96)** | **1.57 (1.26 – 1.95)** |
| Male |  |  |  |  |
| Not religious at all | reference | reference | reference | reference |
| Slightly religious | 0.95 (0.80 - 1.13) | 0.98 (0.83 - 1.17) | 1.01 (0.85 - 1.20) | 1.01 (0.85 - 1.21) |
| Moderately religious | 1.04 (0.86 – 1.26) | 1.09 (0.90 – 1.32) | 1.12 (0.92 – 1.36) | 1.13 (0.93 – 1.37) |
| Extremely religious | 0.91 (0.69 – 1.19) | 0.93 (0.70 – 1.22) | 0.95 (0.72 – 1.25) | 0.95 (0.72 – 1.25) |

Model 1 was adjusted for age and sex. Model 2 wad adjusted for health habits (smoking, alcohol consumption, and exercise) and body mass index in addition to the covariates in Model 1. Model 3 was adjusted for marital status in addition to the covariates in Model 2. Model 4 was adjusted for medical history (current hypertension, diabetes, dyslipidemia, and any cancer, and any past cancer) in addition to the covariates in Model 3.

Numbers in bold indicate p <0.05.
